# Intravitreal ranibizumab improves macular sensitivity in patients with central retinal vein occlusion and macula edema

**DOI:** 10.1186/s12886-022-02478-9

**Published:** 2022-06-04

**Authors:** Taro Otawa, Hidetaka Noma, Kanako Yasuda, Akitomo Narimatsu, Masaki Asakage, Akina Tanaka, Hiroshi Goto, Masahiko Shimura

**Affiliations:** 1 Department of Ophthalmology, Hachioji Medical Center, Tokyo Medical University, Tatemachi 1163, 193-0998 Hachioji, Tokyo, Japan; 2 Department of Ophthalmology, Tokyo Medical University, Tokyo, Japan

**Keywords:** Central retinal vein occlusion, Macular edema, Ranibizumab, Macular sensitivity

## Abstract

**Background:**

Patients with central retinal vein occlusion (CRVO) and macular edema often are treated by intravitreal ranibizumab injection (IRI). The role of changes in macular sensitivity in the positive effects of IRI on visual functions is unclear. Therefore, we assessed the relationship between macular sensitivity and improvement of visual functions.

**Methods:**

We included 15 eyes of 15 patients with treatment-naïve CRVO and followed patients for 6 months after pro re nata IRI. IRI was repeated if the central macular thickness was greater than or equal to 300 µm. Microperimetry-3 was used to measure macular sensitivity within the central 1-mm, 3-mm, and 6-mm fields before and monthly for 6 months after IRI.

**Results:**

IRI significantly improved mean macular sensitivity over time within the central 1-mm, 3-mm, and 6-mm fields (all *P* < 0.001). None of the fields showed significant differences in the change of mean macular sensitivity between patients with little improvement in best corrected visual acuity (BCVA; i.e., in patients with a change in logarithm of the minimum angle of resolution [logMAR] BCVA < 0.3) and those with marked improvement in BCVA (change in logMAR BCVA > 0.3). The mean macular sensitivity before IRI showed correlations with the improvement of macular sensitivity in every field.

**Conclusion:**

These findings suggest that IRI improves macular sensitivity in patients with CRVO and macular edema independent of any improvement in BCVA and that macular sensitivity before treatment is associated with improvement of macular sensitivity after treatment.

## Background

Central retinal vein occlusion (CRVO) and branch retinal vein occlusion (BRVO) are frequently found in people with type 2 diabetes, hypertension, hyperlipidemia, and arteriosclerosis, among other diseases. A common feature of CRVO and BRVO is macular edema, which causes visual impairment. Vascular occlusion research showed that vascular endothelial growth factor (VEGF) is associated with macular edema in CRVO [1], and anti-VEGF injection is used to treat macular edema in patients with CRVO and BRVO. Several randomized clinical trials have demonstrated better visual prognosis with repeated intravitreal anti-VEGF treatment [2–4].

Once macular edema has resolved, visual acuity returns to a relatively good level in most patients. However, visual functions can remain poor in some patients and affect reading speed [5] and contrast sensitivity [6], for example. Therefore, besides visual acuity, visual functions should also be assessed after treatment for macular edema, and studies have found that retinal sensitivity, in particular macular sensitivity, is suitable for evaluating visual function [7, 8].

Some research has been performed on macular sensitivity after anti-VEGF therapy for macular edema in CRVO and BRVO [9, 10]. However, it remains unclear whether macular sensitivity plays a role in improvement of visual functions after treatment of macular edema. Therefore, we evaluated macular sensitivity in patients with CRVO before and after intravitreal injection of the anti-VEGF ranibizumab and examined the relationship between macular sensitivity and improvement of visual functions.

## Methods

### Subjects

This retrospective study was performed in consecutive patients with treatment-naïve, non-ischemic CRVO and visual impairment due to macular edema who underwent intravitreal ranibizumab injection (IRI) at the Department of Ophthalmology, Hachioji Medical Center, Tokyo Medical University, Tokyo, Japan. Inclusion criteria were as follows: no history of intravitreal injections of anti-VEGF or other drugs; no previous treatment with intraocular corticosteroids, retinal photocoagulation, pars plana vitrectomy, or laser; age over 30 years; less than 3 months between symptom onset and initial examination; logarithm of the minimum angle of resolution (logMAR) best corrected visual acuity (BCVA) between 0.15 and 1.3; central macular thickness (CMT) greater than 300 μm as measured by optical coherence tomography (OCT); and follow-up of at least 6 months. Exclusion criteria were ischemic CRVO, defined as at least 10 disc areas of capillary non-perfusion [11]; macular ischemia; diabetic retinopathy due to type 2 diabetes; coexisting ocular disease (i.e., epiretinal membrane or glaucoma); and systemic disorders other than hypertension or hypercholesterolemia. After patients had provided informed consent, IRI was performed via the pars plana with a 30-gauge needle, 3.5 mm posterior to the limbus.

We included 15 eyes of 15 patients (9 men, 6 women) who had macular edema due to CRVO and had been followed up for 6 months after their initial treatment. The mean (SD) age of the sample was 58.5 (11.9) years. In all patients, CRVO had been diagnosed by fundus and spectral-domain OCT (SD-OCT) examinations. After diagnosis, all patients were evaluated monthly and treated with IRI (Lucentis; 0.5 mg in 0.05 ml; Genentech, Inc., South San Francisco, CA) pro re nata. IRI was repeated if the central macular thickness, including any serous retinal detachment [SRD], was greater than or equal to 300 µm. The number of additional IRI injections was recorded as a measure of recurrence.

The study was performed in accordance with the Declaration of Helsinki and was approved by the Institutional Research Board of Hachioji Medical Center, Tokyo Medical University.

### Functional mapping by microperimetry

**Fig. 1:**
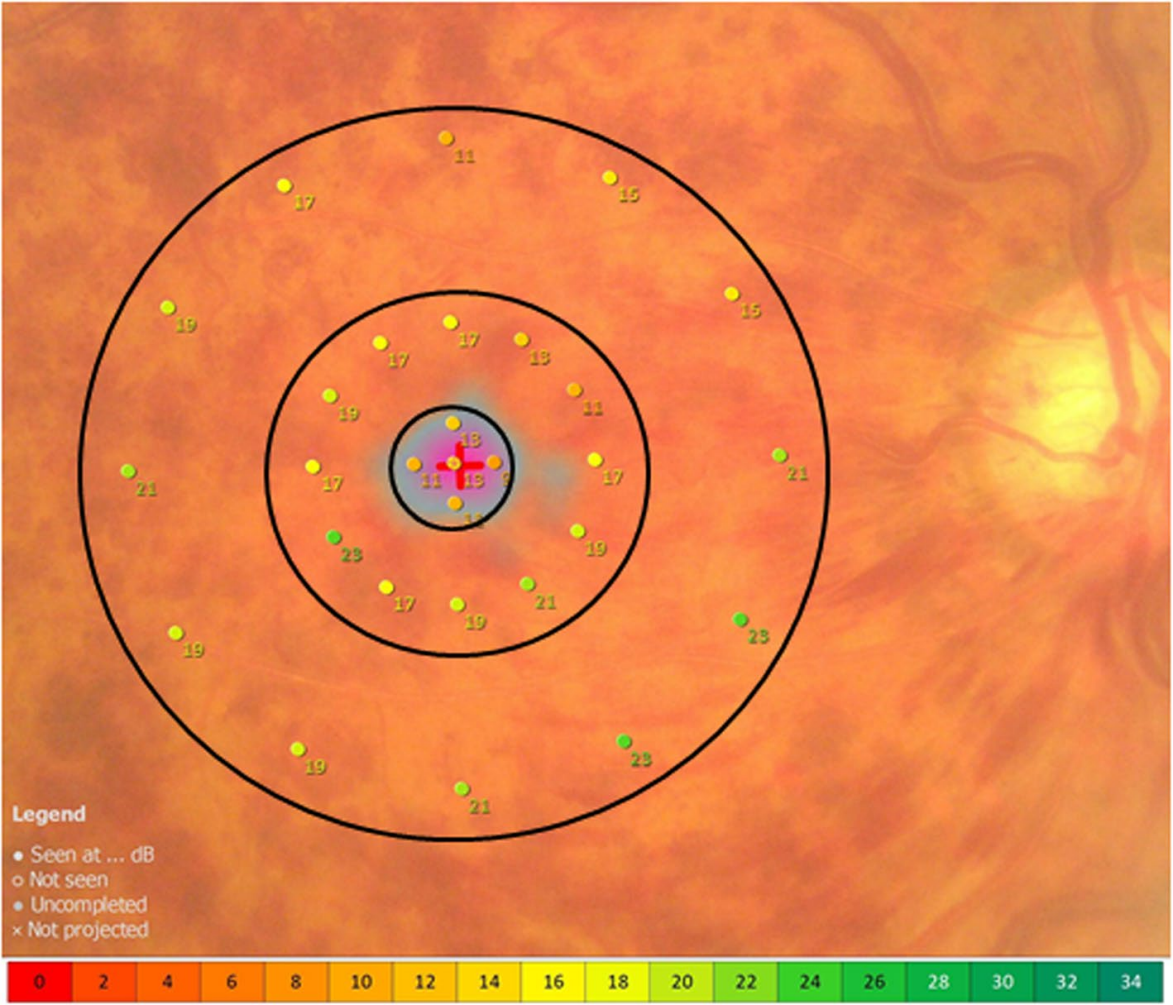
Measurement of retinal sensitivity by microperimetry. The figure shows a typical macular sensitivity map obtained with the MP-3 system. The mean macular sensitivity was calculated within the central 1-mm, 3-mm, and 6-mm fields. The MP-3 system tested the foveal region (central 1-mm field) at 5 points and the macular regions (central 3-mm and 6-mm fields) at 17 points and 29 points, respectively

We assessed functional mapping by microperimetry at baseline and once a month until 6 months after IRI. Microperimetry combines digital fundus imaging with automated perimetry and was performed with the MP-3 microperimeter (Nidek, Gamagori, Japan). In all patients, the pupil diameter was larger than 4 mm, as is required for measurements with the MP-3. The MP-3 measurement was carried out with the 4–2 full threshold staircase strategy and the standard Goldmann III stimulus size on a background luminance of 31.4 asb. The maximum luminance of the MP-3 is 10,000 asb, which results in a stimulus dynamic range of 0 to 34 dB. The size of the fixation target was adjusted according to the patient’s visual acuity. One of the advantages of the MP-3 perimeter is that it automatically compensates for any refractive error in the patient’s eye [12]. Retinal sensitivity maps were obtained with the MP-3 program, which examines the central 20 degrees of the macula and uses a different number of stimulus locations depending on the area being investigated. Macular sensitivity within the central 1 mm was defined as the mean retinal sensitivity of 5 stimulus locations; macular sensitivity within the central 3 mm, as the mean retinal sensitivity of 17 stimulus locations; and macular sensitivity within the central 6 mm, as the mean retinal sensitivity of 29 stimulus locations (Fig. 1). The central 1-mm field corresponded to the foveal region, and the central 3-mm and 6-mm fields, to the macular region.

### Clinical parameters

All patients underwent a comprehensive ocular examination before the start of the study (baseline) and at every follow-up visit, i.e. at 1, 2, 3, 4, 5, and 6 months after IRI. BCVA was measured as the decimal visual acuity by using the Landolt Chart and converted to the logMAR. Fluorescein angiography (Heidelberg Engineering, Heidelberg, Germany) was performed at the baseline visit to determine the area of capillary non-perfusion. At the follow-up examinations, visual acuity was measured with the logMAR chart (5 m; NEITZ LVC-10, Tokyo, Japan) and CMT was measured with Spectralis OCT (Heidelberg Engineering, Heidelberg, Germany). The Spectralis OCT mapping images were generated with the currently available Spectralis software. CMT was defined as the thickness of the central 1-mm field on the OCT mapping image and as the distance between the inner limiting membrane and the retinal pigment epithelium (including any SRD) and was automatically measured by the computer software. We used the images obtained with OCT to quantify CMT and evaluated the presence of SRD.

### Changes in clinical parameters

To assess improvement of vision, we calculated the change in BCVA over 6 months by subtracting the value at the 6-month follow-up from the value at baseline (i.e., before IRI). To assess macular sensitivity, we calculated the percentage changes in macular sensitivity (%ΔMS), as follows: %ΔMS = (MS_pre_—MS_6 months_)/MS_pre_ × 100 = (1—MS_6 months_/MS_pre_) × 100, where MS_pre_ and MS_6 months_ represent the sensitivity values at baseline and 6 months after IRI, respectively.

### Statistical analysis

All analyses were performed with SAS System 9.4 software (SAS Institute Inc., Cary, North Carolina, USA). Results are presented as the mean ± SD or as the frequency. We compared unpaired continuous variables with an unpaired Student’s t test and paired continuous variables with a paired Student’s t test. One-way or two-way repeated measures analysis was performed with the use of mixed model for evaluating the macular sensitivity. Two-tailed *P* values of less than 0.05 were considered to indicate statistical significance.

## Results

### Clinical characteristics

Table 1 shows the clinical characteristics, visual functions, and OCT parameters before and 6 months after IRI. The mean number of injections in 6 months was 2.7 ± 1.4; 7 patients had 1 or 2 injections, and 8 had 3 or more injections. Eleven patients had SRD before treatment, but only 4 of these patients had it 6 months after treatment. BCVA and CMT improved significantly after IRI (*P* = 0.018 and *P* = 0001, respectively). The presence of SRD before IRI was not significantly correlated with the number of injections or the type of injection regimen (1–2 injections or 3 or more injections), but the presence of SRD 6 months after IRI was significantly correlated with both the number of injections and the type of injection regimen (*P* = 0.026 and *P* = 0.029, respectively).

**Table 1:** Clinical characteristics and macular sensitivity before and after intravitreal ranibizumab injection in patients with central retinal vein occlusion and macular edema

	Before treatment	6 months after treatment	*P* value
Age (years)	58.5 ± 11.9^a^		
Gender (female/male)	6/9		
Duration of macular edema (days)	34.5 ± 23.4^a^		
Hypertension	11		
Systolic Blood pressure (mmHg)	138 ± 11.3^a^		
Diastolic Blood pressure (mmHg)	87.1 ± 10.3^a^		
Hyperlipidemia	7		
Number of injections		2.7 ± 1.4^a^	
Injection type (1–2 injections/3 or more injections)		7/8	
BCVA (logMAR)	0.52 ± 0.39^a^	0.25 ± 0.33^a^	0.018
Central macular thickness (μm)	721 ± 207^a^	402 ± 209^a^	0.001
Macular sensitivity within 1 mm (dB)	12.8 ± 5.68^a^	19.2 ± 6.74^a^	0.007
Macular sensitivity within 3 mm (dB)	16.2 ± 5.39^a^	21.8 ± 4.82^a^	0.002
Macular sensitivity within 6 mm (dB)	17.5 ± 5.16^a^	22.2 ± 4.25^a^	< 0.001
Presence of SRD	11	4	0.011

*BCVA* Best-corrected visual acuity, *log MAR* Logarithm of the minimum angle of resolution, ^a^Mean ± standard deviation (SD)

**Fig. 2:**
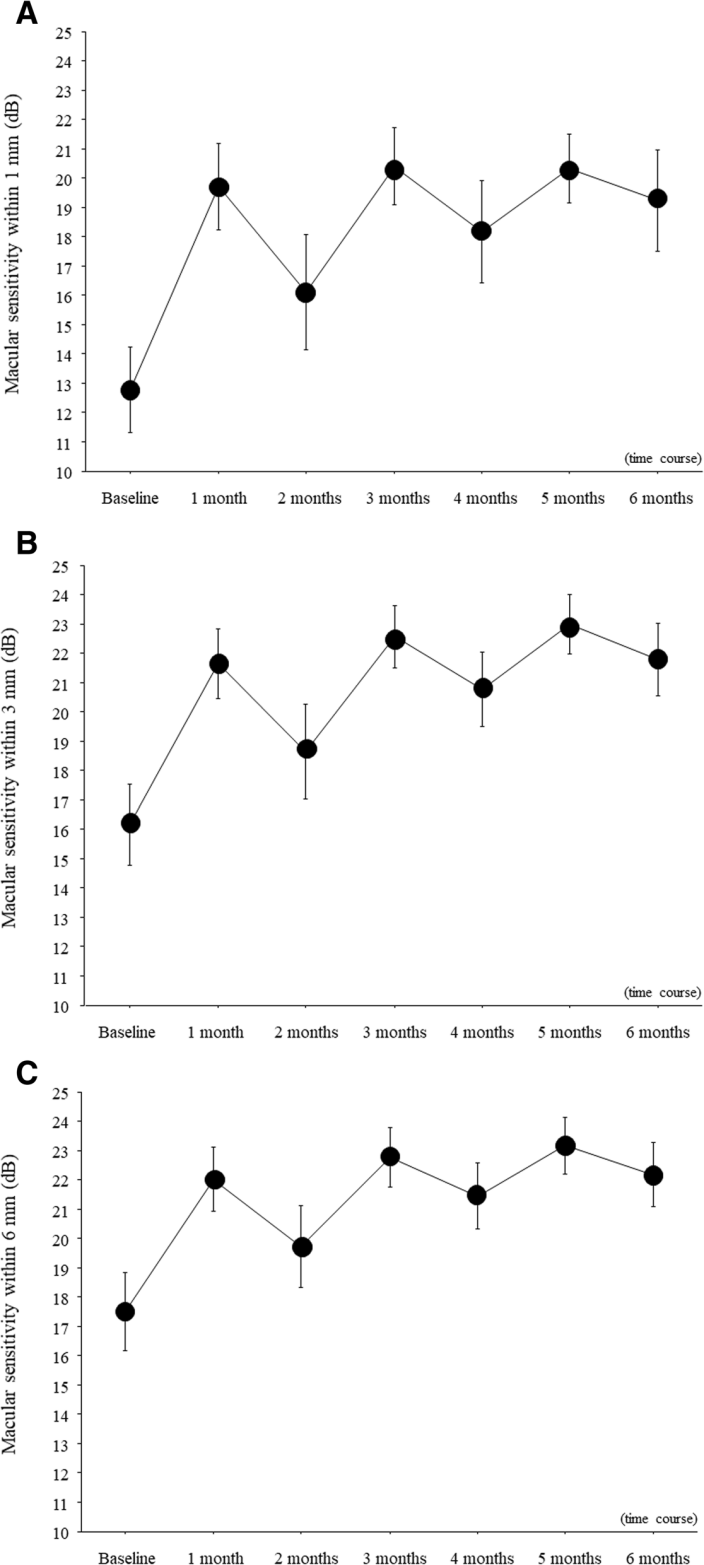
Trend profile of mean macular sensitivity after intravitreal ranibizumab injection. In the 3 fields examined by microperimetry (central 1-mm, 3-mm, and 6-mm fields), mean macular sensitivity significantly improved 6 months after intravitreal ranibizumab injection (IRI) in patients with central retinal vein occlusion. **A** Significant improvement in the central 1-mm field (*P* < 0.001). **B** Significant improvement in the central 3-mm field (*P* < 0.001). **C** Significant improvement in the central 6-mm field (*P* < 0.001)

**Fig. 3:**
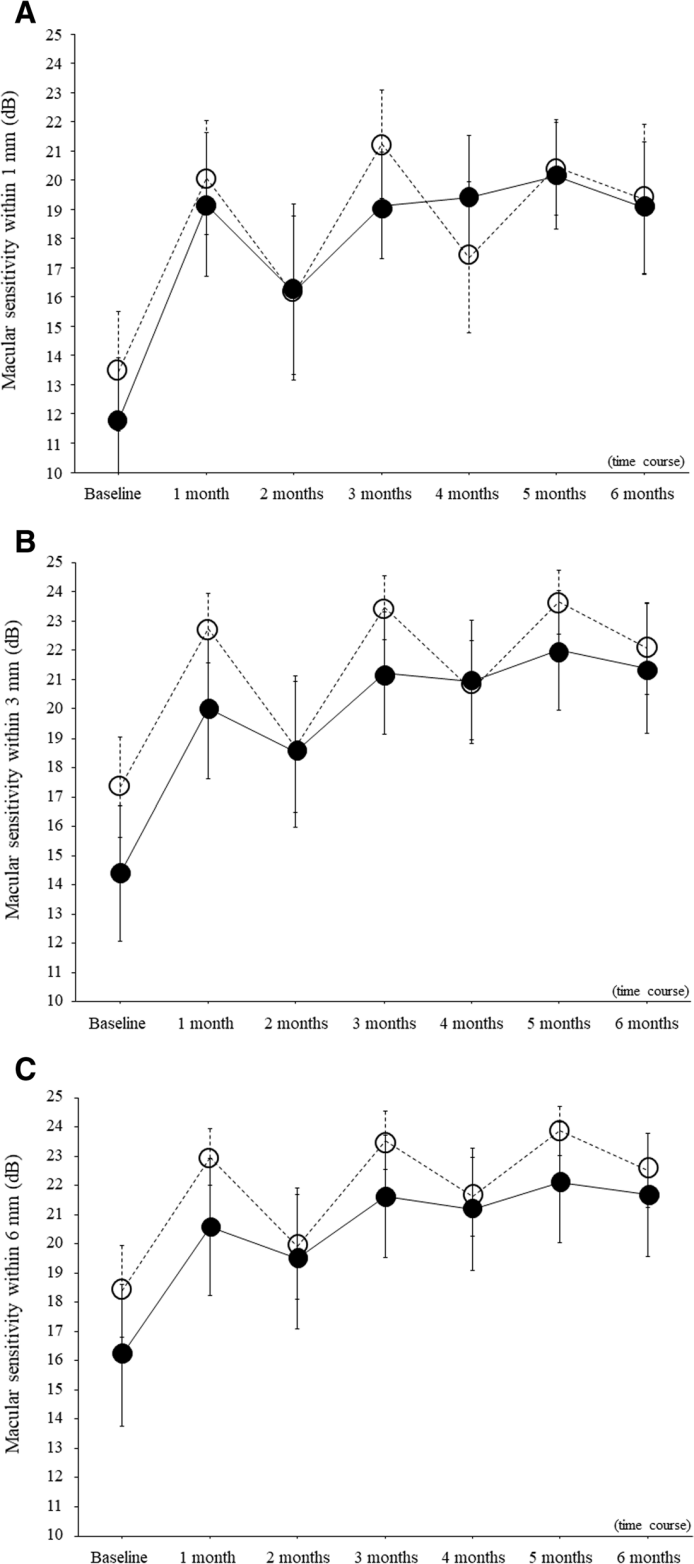
Trend profile of mean macular sensitivity after intravitreal ranibizumab injection between patients with little improvement in best corrected visual acuity (change in logMAR BCVA < 0.3) and those with marked improvement in best corrected visual acuity (change in logMAR BCVA > 0.3). The trend profile of mean macular sensitivity after intravitreal ranibizumab injection showed no significant difference between patients with little improvement in BCVA (dotted line) and those with marked improvement in BCVA (solid line) in any of the 3 fields examined by microperimetry (central 1-mm, 3-mm, and 6-mm fields) **A** Results in the central 1-mm field (*P* = 0.830). **B** Results in the central 3-mm field (*P* = 0.551). **C** Results in the central 6-mm field (*P* = 0.746)

Figure 2 shows the monthly change in macular sensitivity from baseline to 6 months after IRI. Over time, mean macular sensitivity significantly improved within the central 1-mm, 3-mm, and 6-mm fields (all *P* < 0.001; Figs. 2A-C). We found no significant differences in the changes of mean macular sensitivity within the central 1-mm, 3-mm, and 6-mm fields between patients with little improvement in BCVA (change in logMAR BCVA < 0.3) and those with marked improvement in BCVA (change in logMAR BCVA > 0.3; 1-mm field, *P* = 0.830; 3-mm field, *P* = 0.551; and 6-mm field, *P* = 0.746; Figs. 3A-C).

### Correlations of visual functions with BCVA, macular sensitivity, and CMT

Table 2 shows the correlations of BCVA, macular sensitivity, and CMT before and after IRI with the improvement of visual functions at 6 months after IRI. BCVA at baseline was significantly correlated with the improvement of BCVA (*P* < 0.05); mean macular sensitivity in the central 1-mm at baseline was significantly correlated with the improvement of macular sensitivity in the central 1-mm, 3-mm, and 6-mm fields (*P* < 0.05, *P* < 0.001, and *P* < 0.001, respectively); mean macular sensitivity in the central 3-mm at baseline was significantly correlated with the improvement of macular sensitivity in the 1-mm, 3-mm, and 6-mm fields (*P* < 0.05, *P* < 0.001, and *P* < 0.001, respectively); mean macular sensitivity in the central 6-mm at baseline was significantly correlated with the improvement of macular sensitivity in the 1-mm, 3-mm, and 6-mm fields (*P* < 0.05, *P* < 0.001, and *P* < 0.001, respectively); and CMT at baseline was significantly correlated with the improvement of macular sensitivity in the 3-mm and 6-mm fields (both *P* < 0.05, respectively).

**Table 2:** Correlations between improvement of visual functions after treatment and ocular parameters in patients with central retinal vein occlusion

Parameters	Improvement of BCVA	Improvement of macular sensitivity within 1 mm	Improvement of macular sensitivity within 3 mm	Improvement of macular sensitivity within 6 mm
	*P* value (correlation coefficient)	*P* value (correlation coefficient)	*P* value (correlation coefficient)	*P* value (correlation coefficient)
*Before IRI*				
Duration of macular edema, days	0.689 (-0.11)	0.502 (-0.19)	0.355 (-0.26)	0.301 (-0.29)
BCVA (logMAR)	0.002 (0.72)	0.942 (0.02)	0.136 (0.40)	0.100 (0.44)
Macular sensitivity within 1 mm, dB	0.344 (-0.26)	0.005 (-0.68)	< 0.001 (-0.80)	< 0.001 (-0.82)
Macular sensitivity within 3 mm, dB	0.159 (-0.38)	0.011 (-0.64)	< 0.001 (-0.84)	< 0.001 (-0.87)
Macular sensitivity within 6 mm, dB	0.221 (-0.34)	0.022 (-0.59)	< 0.001 (-0.81)	< 0.001 (-0.85)
Central macular thickness, μm	0.093 (0.45)	0.059 (0.50)	0.005 (0.68)	0.007 (0.66)
Presence of SRD	0.434	0.700	0.999	0.952
*6 months after IRI*				
Central macular thickness, μm	0.036 (-0.55)	0.139 (-0.40)	0.172 (-0.37)	0.233 (-0.33)
Presence of SRD	0.728	0.206	0.208	0.255
Number of injections	0.075 (-0.47)	0.457 (-0.21)	0.407 (-0.23)	0.497 (-0.19)
Injection type	0.071	0.905	0.597	0.667

*Bcva* Best-corrected visual acuity, *IRI* Intravitreal ranibizumab injection, *log MAR* Logarithm of the minimum angle of resolution, *SRD* Serous retinal detachment

In addition, CMT at 6 months after IRI showed a significant correlation with the improvement of BVCA (*P*<0.05). The number of injections (1 or 2 injections or 3 or more injections) and type of injections also were not significantly correlated with any changes in visual functions.

## Discussion

To evaluate whether macular sensitivity plays a role in changes in visual functions after treatment of macular edema, this study evaluated macular sensitivity in patients with CRVO before and after IRI. Regarding visual functions, we found that not only visual acuity but also macular sensitivity within the central 1-mm, 3-mm, and 6-mm fields improved significantly after IRI. This finding suggests that, after anti-VEGF treatment, not only the fovea but also the larger macular area should be evaluated because treatment improves edema of the entire macula in CRVO. Furthermore, it indicates that—when assessing the visual prognosis of CRVO patients with macular edema—it may be relevant to evaluate not only visual acuity (an indicator of foveal function) but also macular sensitivity (an indicator of retinal sensitivity in the larger macular area). Thus, microperimetry with the MP-3 may demonstrate the effectiveness of anti-VEGF treatment for macular edema associated with CRVO more accurately than measurements of visual acuity alone. This hypothesis is supported by our earlier finding that retinal thickness and retinal volume are related to visual acuity and retinal sensitivity in patients with CRVO and macular edema [8].

Interestingly, we found no significant differences in the changes of mean macular sensitivity within the central 1-mm, 3-mm, and 6-mm fields between patients with little improvement in BCVA and those with marked improvement. This result suggests that improvement in macular sensitivity is independent of improvement in visual acuity. Some studies indicated that macular sensitivity in patients with age-related macular degeneration and retinitis pigmentosa is associated with vision-related quality of life [13, 14]. Our result that macular sensitivity improved even in patients with little improvement in visual acuity may help to explain these earlier findings. Furthermore, we found no significant correlations between the number of injections (as a measure of recurrence) and the improvement of BCVA and macular sensitivity, suggesting that recurrence did not influence the improvement of BCVA and macular sensitivity.

We also found that BCVA before IRI was significantly correlated with the improvement of BCVA, that macular sensitivity before IRI was significantly correlated with the improvement of macular sensitivity in all 3 central fields and that CMT before IRI was significantly correlated with the improvement of macular sensitivity in the 3-mm and 6-mm fields. These findings suggest that treatment improved visual functions in patients with poor baseline values for BCVA, CMT, and/or macular sensitivity. On the other hand, CMT at 6 months after IRI was significantly correlated with improvement of BCVA but not with the improvement of macular sensitivity, suggesting that improvement of CMT after IRI does not affect improvement of macular sensitivity but does affect improvement of BCVA. A recent study found that resolution of subretinal fluid 6 months after anti-VEGF treatment was associated with improved retinal sensitivity [15], indicating that once macular edema improves, macular sensitivity may be less likely to worsen.

This study was limited by the small sample size, which meant that the repeated measures analyses were underpowered. Consequently, the clinical relevance of the study findings should be interpreted with caution, and large, prospective studies are required on macular sensitivity after anti-VEGF therapy in patients with macular edema due to CRVO.

## Conclusions

This study demonstrated that, over 6 months, IRI significantly improves mean macular sensitivity within the central 1-mm, 3-mm, and 6-mm fields in patients with CRVO and macular edema and that changes in mean macular sensitivity show no significant differences between patients with little improvement in BCVA and those with marked improvement. Furthermore, mean macular sensitivity before IRI shows significant correlations with improvement of macular sensitivity in the 3 fields mentioned above. These findings suggest that IRI improves macular sensitivity independent of any improvement in BCVA and that macular sensitivity before treatment is associated with improvement of macular sensitivity. Because improvements in macular sensitivity may increase quality of life even in patients who show no improvement in BCVA, these findings are of great relevance in clinical practice and warrant further evaluation in prospective studies in larger samples.

## Data Availability

The data that support the findings of this study are not publicly available because they contain information that could compromise the privacy of research participants and because ethics committee approval for release of the data was not obtained.

## Abbreviations

BCVA: Best-corrected visual acuity
BRVO: Branch retinal vein occlusion
CMT: Central macular thickness
CRVO: Central retinal vein occlusion
IRI: Intravitreal ranibizumab injection
LogMAR: Logarithm of the minimum angle of resolution
OCT: Optical coherence tomography
SD-OCT: Spectral-domain OCT
SRD: Serous retinal detachment
VEGF: Vascular endothelial growth factor
